# The impact of TMS‐enhanced cognitive control on forgiveness processes

**DOI:** 10.1002/brb3.2131

**Published:** 2021-03-30

**Authors:** Moritz J. Maier, David Rosenbaum, Martin Brüne, Andreas J. Fallgatter, Ann‐Christine Ehlis

**Affiliations:** ^1^ Center for Responsible Research and Innovation at the Fraunhofer IAO Berlin Germany; ^2^ Psychophysiology and Optical Imaging Department of Psychiatry and Psychotherapy University of Tuebingen Tuebingen Germany; ^3^ Department of Psychiatry, Psychotherapy and Preventive Medicine Division of Cognitive Neuropsychiatry and Psychiatric Preventive Medicine LWL University Hospital Bochum Ruhr‐University Bochum Bochum Germany; ^4^ Werner Reichardt Centre for Integrative Neuroscience (CIN) Tuebingen Germany; ^5^ LEAD Graduate School & Research Network University of Tuebingen Tuebingen Germany

**Keywords:** cognitive control, emotion regulation, fNIRS, revenge, TMS

## Abstract

**Background:**

Cognitive control is thought to be necessary for forgiveness processes.

**Materials and Methods:**

To examine this correlation, highly impulsive participants, who often fail to inhibit feelings of revenge, received activating theta burst stimulation (TBS) of a classical cognitive control region of the brain, the right dorsolateral prefrontal cortex (rDLPFC). For testing forgiveness ability participants received verum TBS versus sham TBS in a randomized, double‐blinded, within‐subjects design. In both sessions, they first learned that there are fair and unfair opponents in an ultimatum game, and subsequently played a dictator game with reversed roles with the option to revenge or forgive the opponents from the previous game.

**Results:**

Contrary to our hypothesis, activating TBS did not increase forgiving behavior toward unfair opponents. However, it increased the generosity toward previously fair opponents.

**Conclusion:**

As an explanation it is discussed that the TBS can only affect “cold” emotions such as greed, but not the “hot” emotions such as anger.

## Graphical abstract

The results of this study provide exciting insights into forgiveness processes in general but especially into the connection between cognitive control, emotional foundations of behavior and forgiveness and the mode of action of neuromodulation.

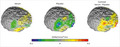

## 1 INTRODUCTION

The ability to forgive others for their misdemeanors is highly relevant to cooperation and reciprocity (Trivers, 1971). In addition, individuals with better forgiveness ability show higher rates of well‐being (Worthington et al., 2007), better cardiovascular health (Friedberg et al., 2007), better relationship quality (Allemand et al., 2007), and lower mortality (Toussaint et al., 2012). This suggests that forgiveness is positively associated with many areas of life.

Approaches to the definition of the phenomenon of forgiveness have focused on different elements involved in the process of forgiving (McCullough, 2001; Riek & Mania, 2012). For example, McCullough et al. (1997) define forgiveness as a change in motivation, moving away from avoiding contact with the offender and seeking revenge toward more conciliatory behavior. Similarly, Worthington and Wade (1999) describe forgiveness as the replacement of negative emotions with positive emotions toward the offender. Finally, Wilkowski et al. (2010) characterize forgiveness as a two‐step process involving (1) the decision to forgive and (2) inhibition of revenge. This points to a central role of cognitive control in forgiving behavior.

Cognitive control as a neuropsychological concept consists of three subfunctions: task switching, updating, and inhibition (Miyake et al., 2000). Cognitive control represents a highly relevant function for almost all areas of life, including academic or financial success (Moffitt et al., 2011). A lack of cognitive control, in contrast, has been associated with various mental disorders (e.g. Barth et al., 2015; Ehlis et al., 2008; Fallgatter et al., 2005; Rosenbaum et al., 2018).

At the neuronal level, neuroimaging studies have shown that response conflicts requiring increased levels of cognitive control are associated with increased activity in the anterior cingulate cortex (ACC). This internal monitoring system reports potential conflicts that inhibit prepotent automatic responses to the dorsolateral prefrontal cortex (DLPFC), which subsequently implements cognitive control to resolve the response conflict (Conflict Monitoring Theory; Botvinick et al., 2001, 2004; Egner & Hirsch, 2005; MacDonald et al., 2000; Milham et al., 2001). To investigate the correlation between forgiveness processes and DLPFC activation, Brüne et al. (2013) combined an ultimatum game and a dictator game in an fMRI study. First, participants played an ultimatum game in which they had to accept or reject fair or unfair offers from the opponents, who had to split 10 Euro in each trial. In this game, participants implicitly learned that there are fair (offers between € 3 and € 5) und unfair (offers between € 0 and € 2) opponents. Then the roles were reversed and the participants had to split the money in a dictator game. This gave the participants the opportunity to revenge by allocating an unfair amount to the unfair opponents or to forgive by allocating a fair amount of money. Forgiveness behavior here was associated with higher activation in the right DLPFC, which is consistent with the conflict monitoring theory outlined above. To further investigate this relationship, Maier et al. (2018) combined the gaming paradigm of Brüne et al. (2013) with inhibitory continuous theta burst stimulation (cTBS; Gehan, 1965) of the right DLPFC. With cTBS, the activation of a specific brain area can be reduced for a certain time (Huang et al., 2005). According to the conflict monitoring theory and the results of Brüne et al. (2013), lower rates of forgiveness were observed with reduced activity in the right DLPFC (compared to placebo condition involving sham stimulation). Since a causal involvement of the right DLPFC in forgiveness processes is suspected, the question arises if a targeted *increase* in activation of the right DLPFC (induced by transcranial magnetic stimulation (TMS)) could also influence forgiveness processes in the opposite direction (i.e., toward an increase in forgiveness).

Since impulsivity is negatively correlated with cognitive control, highly impulsive individuals might benefit from stimulation of the right DLPFC by gaining more cognitive control over their prepotent emotional responses to unfairness. This could also be clinically relevant, as impulsivity and poor inhibitory control are associated with several mental disorders such as ADHD or borderline personality disorder (Bari & Robbins, 2013; Christodoulou et al., 2006; Ehlis et al., 2008; Herrmann et al., 2009). Accordingly, we aimed to investigate the effect of intermittent TBS (iTBS; 24) over the right DLPFC on forgiveness behavior in a highly impulsive group of participants. iTBS has the potential to increase neuronal activity in the targeted brain area for at least 15 min (Huang et al., 2005). For this purpose, we applied iTBS in a within‐subject‐design, a double‐blind placebo‐controlled experiment in randomized order. To control for the stimulation effect and investigate the underlying neuronal processes, functional near‐infrared spectroscopy (fNIRS) was performed over the DLPFC.

Specifically, we hypothesized that the stimulation of the right DLPFC increases forgiving behavior by improving cognitive control and reduces the effect of prepotent impulsive emotional responses. We also expected an increase in activation in the right DLPFC (measured by fNIRS) in the verum condition compared with placebo.

## 2 Methods

### 2.1 Participants

Participants were recruited via a university‐wide circular email. Included in this email was a link to the impulsivity scale of the Adult ADHD Self‐Report Scale (ASRS; Kessler et al., 2005). Only participants with scale scores between 15 and 30 were contacted for this study, as these scores indicate high levels of impulsivity. Further exclusion criteria were chronic or acute diseases potentially affecting the cerebral metabolism (craniocerebral trauma, kidney failure, diabetes, uncontrolled hypertension), neurological or psychiatric disease (present or past), acute endangerment of self or others and any contraindications for TMS (see Jasper, 1958). A total of 30 subjects participated in the study, all of whom were students at the University of Tübingen. The required group size was determined using G‐Power (two tails, Wilcoxon signed‐rank test (matched pairs), normal distribution (Faul et al., 2009), assuming a large effect (0.7, Cohen, 1988), based on a previous study with the similar paradigm (Maier et al., 2018). The average age was 23.75 years (*SD* = 3.05); 5 participants were male. Participants received financial compensation of 10 Euro per hour for their participation. The study was in accordance with the current version of the Declaration of Helsinki and was approved by the Ethics Committee of the Medical Faculty of the University of Tübingen. Written informed consent was obtained from all participants. The different questionnaire scores are described in Table 1.

**TABLE 1:** Questionnaire scores of the study sample

Questionnaire	Mean	*SD*
ASRS	20.41	5.29
BDI	5.86	5.61
Willingness to forgive	21.20	4.34
Tendency for Forgiveness	14.36	4.24

Note

Adult ADHD self‐report scale (ASRS; Impulsivity Scale; Kessler et al., 2005), Beck Depression Inventory (BDI; Faul et al., 2009), Willingness to Forgive Scale (Allemand et al., 2008), Tendency to Forgive Scale (Brown, 2003).

### 2.2 Paradigm

The paradigm was adapted from studies of Brüne et al. (2013) and Maier et al. (2018). It consists of two consecutive tasks, an ultimatum game followed by a dictator game. Each game comprises 40 trials and has a duration of approximately 9 min. In the ultimatum game, participants are first presented with a picture and the name of the opponent in the trial for 3 s. The presentation of the current opponent was followed by a break with a jittered fixation cross for 2–3 s. The participant was then presented with the offer of the current opponent. Fictitious 10 Euro were split in each trial by a total of 4 opponents: 2 unfair (1 male, 1 female; offers between 0 € and 2 €) and 2 fair (1 male, 1 female; offers between 3 € and 5 €; cf.; Brüne et al., 2013; Sanfey et al., 2003). After the participant decided to accept or reject the offer by pressing a button, a feedback screen with the money allocations was displayed for 3 s. If the participant rejected an offer, the opponent also received 0 €. The participants implicitly learned in this game that there are fair and unfair opponents. After completion of the ultimatum game, participants received the cTBS (described below) in a separate room. Approximately 8 min after finishing the ultimatum game and putting on the fNIRS cap, the dictator game was started. All temporal aspects were the same in the dictator game and in the ultimatum game. Only the roles changed, so that in each trial the participants had to distribute fictitious 10 Euro among the opponents from the previous game. An important difference is that in this game the opponents had no possibility to reject an offer made by the participants, the distribution of money is determined exclusively by generosity and not by strategic thinking. Therefore, in this game, participants had the option to forgive their unfair opponents by allocating a fair amount of money or to revenge against them by allocating an unfair amount of money. Throughout the game, participants were instructed to imagine that they were playing for real money and with real persons. The “Presentation” software package (Neurobehavioral Systems Inc.) was used to present the experiment.

### 2.3 Intermittent theta burst stimulation (iTBS)

The iTBS was applied at electrode position F4 (Herwig et al., 2003) over the right DLPFC using the iTBS protocol of Huang et al. (2005). In general, iTBS shows a lasting effect for at least 15 min after stimulation of 3 min and 10 s. The protocol was used as follows: A 2‐s train of 3 impulses given at 50 Hz was repeated every 10 s for 190 s (600 pulses in total). The impulses were given at 80% of individual motor threshold. Using the MagVenture® active–passive placebo/verum coil system all stimulations were performed in a double‐blind fashion. To ensure similar subjective sensations in both sessions, the stimulated areas of the head were also covered with electrodes in the placebo sessions to induce a feeling comparable to the verum protocol. The experimenter received only a numerical blinded code to start the sessions. To reduce further expectancy effects, all measurements were run by two experimenters. One experimenter only performed the stimulation and hardly talked to the participant. The other experimenter ran the experiments and was in another room during the stimulation. After the fNIRS cap was arranged for approximately 5 min, the DG and the fNIRS measurement began.

### 2.4 fNIRS

Participants' cortical activation during DG was measured using fNIRS. Due to the relative transparency of biological tissue to near‐infrared light and the different absorption spectra of oxygenated (O2Hb) and deoxygenated (HHb) to near‐infrared light (Fallgatter et al., 2004; Haeussinger et al., 2011), it is possible to measure cortical activation through the intact skull. An increase in the concentration of O2Hb and a decrease of HHb indicates cortical activation within the measured brain region. In this study, a commercial multi‐channel NIRS system (ETG‐4000 Optical Topography System; Hitachi Medical Co., Japan) with a temporal resolution of 10 Hz was used. The 3 × 11 probe set with 52 channels, comprising 16 detectors and 17 emitters with an interoptode distance of 3 cm, was placed according to the international 10–20 system for electrode placement (Jasper, 1958). The central optode of the bottom row was placed on Fpz and the bottom row was symmetrically orientated to T3/T4. With the combination of iTBS and fNIRS, it was possible to precisely verify the effect of iTBS.

### 2.5 Questionnaires

In addition to the screening questionnaire, forgiveness and cognitive control related variables were collected using Sosci Survey (Leiner, 2014) within one week before the first measurement. The following questionnaires were Beck Depression Inventory (BDI; Beck et al., 1996), Tendency to Forgiveness Scale (6 statements about forgiveness in general, participants rate their concurrance; Brown, 2003) and the Willingness to Forgive Scale (12 scenarios which include a variety of transgressions and the assesment of the likelihood to forgive; Allemand et al., 2008). These data should ensure that there are no a priori differences between the groups that affect the probability of forgiveness. In both sessions, participants’ desire for revenge, sympathy and perceived fairness (0 to 5, 5 = high feelings of revenge/sympathy/fairness) toward opponents were assessed after the DG.

### 2.6 Analyses

#### 2.6.1 Behavioral data

For the analyses of the behavioral data, the median of the scores was used. We chose the median as this parameter represents the central value of the data is less sensitive to outliers (range mean = 5.025 versus range median = 4.25) and has a lower skewness (skewness mean = −0.375 versus. skewness median=−0.01). Since there is no normal distribution of the values, the nonparametrical Wilcoxon test (Gehan, 1965) was used. To assess a potential interaction effect, first the difference scores (median_fair_opponent_ ‐ median_unfair_opponent_) of the placebo and the verum condition were tested with a Wilcoxon test. Subsequently, two additional Wilcoxon tests were calculated as post hoc tests: the median offer in the placebo versus verum condition toward unfair opponents and the median offer in the placebo versus verum condition toward fair opponents. We used *r* as the effect size (Cohen, 1988).

#### 2.6.2 fNIRS data

The fNIRS data were exported without prior preprocessing, and all analyses were performed with MATLAB 2017 (The MathWorks). All frequencies < 0.01 Hz and > 0.5 Hz were excluded using a bandpass filter. Additionally, the correlation‐based signal improvement procedure (CBSI; Cui et al., 2010) was used to correct motion artifacts. All further analyses were run with the resultant cbsi‐hb. Independent component analysis (ICA; Delorme & Makeig, 2004) was used to exclude residual artifacts. After preprocessing the data, a model‐based analysis for event‐related fNIRS data (Plichta et al., 2007) was applied. The resulting ß values were used for all further tests, which were run using SPSS 22 (SPSS Inc.).

#### 2.6.3 Reaction time

Reaction times of money allocations were analyzed using a 2 × 2 ANOVA with the within‐subjects factors of condition (verum versus placebo) and opponent (fair versus unfair). All trials exceeding two standard deviations from the mean per person were excluded from the analyses. All analyses were performed using SPSS 22 (SPSS Inc.).

## 3 RESULTS

### 3.1 Behavioral results

**FIGURE 1:**
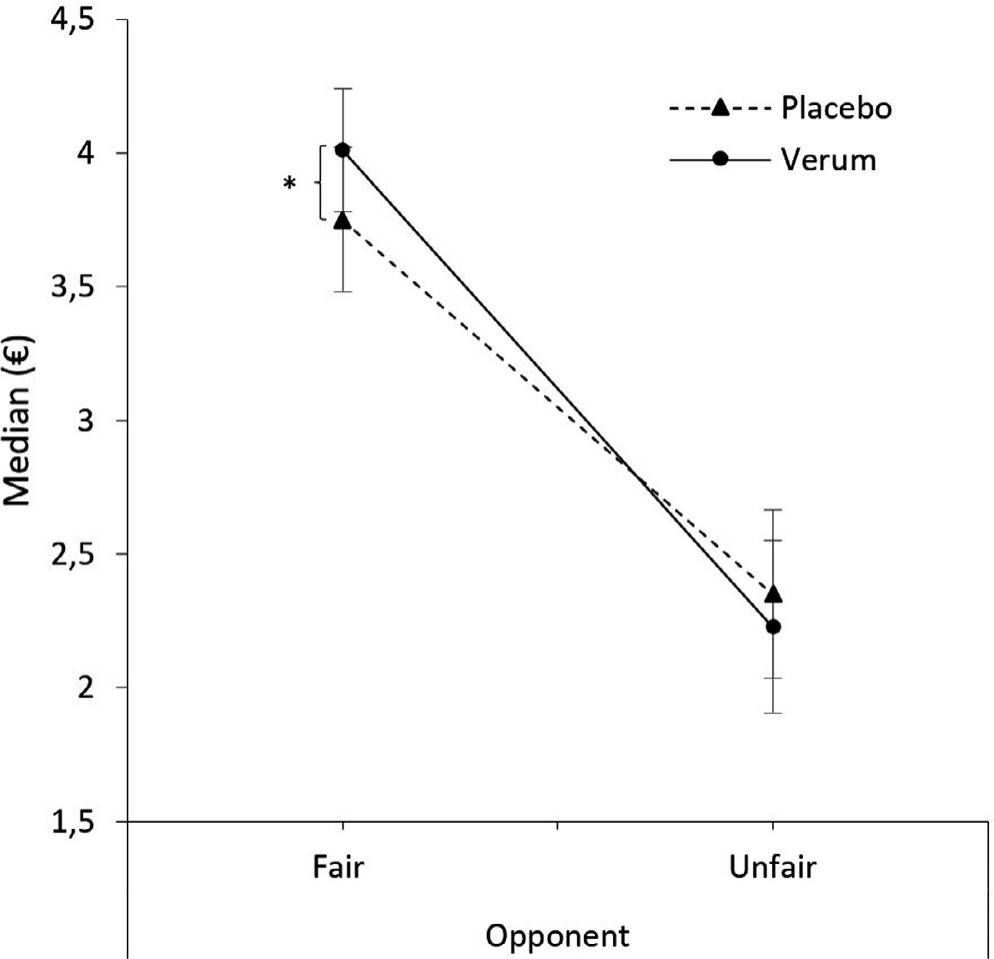
Mean median offer of the subjects in the verum versus placebo condition towards fair versus unfair opponents. The significant difference is marked with a star, the error bars indicate the standard error

In the Wilcoxon test of the difference scores (allocated amount of money; median_fair_opponent_ ‐ median_unfair_opponent_), a significant difference was found between the placebo and verum condition (z = −2.046, *p* = .041, *n* = 30, *r* = .37). This interaction effect is depicted in Figure 1. Contrary to our hypothesis that participants would be more generous toward unfair opponents in the verum condition, we found no differences between the conditions (*z* = −0.941, *p *= .361, *n* = 30, *r* = .16). However, toward fair opponents, we found a significant difference between stimulation conditions (*z* = −2,154, *p *= .031, *n* = 30, *r* = .39) with a higher median after verum (*M* = 4.016, *SD* = 1.262) compared to sham stimulation (*M* = 3.750, *SD* = 1.489).

For reaction times, in a 2 × 2 ANOVA, a main effect of the factor opponent was found (*F*(1,22) = 35.81, *p *< .001), with significantly higher reaction times for unfair opponents (*M* = 3,050 ms, *SD* = 450 ms) than for fair opponents (*M* = 2,907 ms, *SD* = 509 ms).

### 3.2 fNIRS results

**FIGURE 2:**
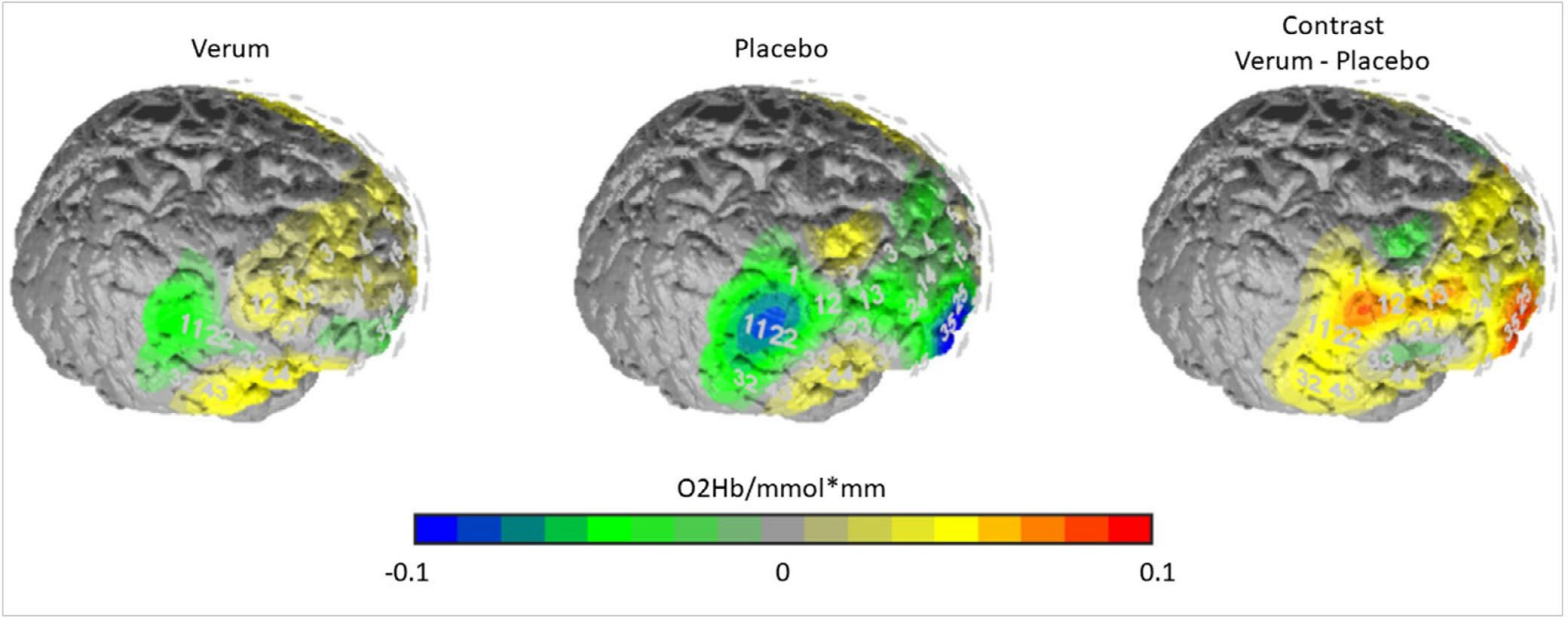
O2hb/mmol*mm for the verum condition, the placebo condition and the contrast verum – placebo for trials with fair offers towards unfair opponents (= forgiveness)

To assess the effect of the activating iTBS on the right DLPFC, brain activation in this area was recorded using fNIRS. In accordance with previous studies (Brüne et al., 2013; Maier et al., 2018), the *forgiveness* condition (with fair offers to previously unfair opponents) was specifically investigated because cognitive control areas (including the right DLPFC) should be most critically involved in this condition. The fNIRS data for the right DLPFC were normally distributed (*p *< .05) according to the Kolmogorov–Smirnov test (Massey, 1951). In a *t*‐test for trials in which forgiveness behavior was shown, the activation in the right DLPFC was significantly higher in the verum condition compared to placebo (*t*(2) = 2.039, *p *= .025). In Figure 2, this difference in the right DLPFC is depicted.

## 4 DISCUSSION

It was hypothesized that participants in the verum condition (increased DLPFC activity through iTBS) would act more in a socially desired manner with respect to money allocations (i.e., closer to 5 Euro), as they would have more resources to apply social norms and resist lower emotional impulses in this condition. We expected more forgiving behavior (fair offers toward previously unfair opponents) in the verum condition. Against our hypothesis, participants in the verum condition are not less revengeful or more forgiving toward previously unfair opponents, even though the analysis of the fNIRS data shows that the activating iTBS was successfully applied over the right DLPFC. A change in behavior was found in the reaction toward previously fair opponents. Here, participants are more generous in the verum condition (= more activity in the right DLPFC) compared to the placebo condition.

The following paragraphs are a first attempt to interpret these surprising results. According to Seuntjens et al. (2015), impulsivity and greed are positively correlated, whereas self‐control and greed are negatively correlated. Thus, greed can be a very relevant motive for individuals with high impulsivity scores and low self‐control. But this motive is often in conflict with general social norms. Greed is regularly associated with negative traits such as selfishness, materialism, dissatisfaction, not be generous, egocentrism, immoral behavior, or arrogance (Seuntjens et al., 2015). Therefore, this motive should be inhibited as much as possible. This requires cognitive control. In the highly impulsive participant group, in which inhibition of unwanted emotions is hampered, an activation of the rDLPFC via iTBS could inhibit the greed motive. It is likely that greed is especially socially sanctioned toward fair persons, so that the activation of the right DLPFC has a greater influence, particularly in this condition.

There could be a difference between trait characteristics such as greed and state emotionality such as anger about a recent transgression. The emotional reactions to unfairness toward oneself are described as very intense emotional feelings of anger (e.g. Civai et al., 2010; Pillutla & Murnighan, 1996). Especially in high arousal states, the amygdala plays a crucial role (Lindquist et al., 2012). The strong correlation of impulsivity and the experience of negative emotions (Boschloo et al., 2013) and its relation to amygdala activity (Gehan, 1965) might explain why in the condition toward unfair opponents the activating TBS of the right DLPFC has no significant influence. The situation is different toward fair opponents, for whom no previous transgression triggers intense negative (“hot”) emotions. Here, the concept of greed might be decisive. It is well described in the literature that the right DLPFC is responsible for the implementation of social norms (e.g. Buckholtz, 2015; Knoch and Fehr, 2007). The results of the present study indicate that the increase of activity in the right DLPFC leads to a stronger implementation of the social norm of generosity (in contrast to greed), but it is not able to influence the “hot” emotion anger, which leads to revenge toward previously unfair opponents. Schaefer et al. (2003) investigated the neural location of “hot” and “cold” processes and point out their differences. In schematic “hot” processes, the ventromedial prefrontal cortex was primarily activated, whereas the anterolateral prefrontal cortex was activated in “cold” propositional situations. Since our TMS coil specifically stimulates the lateral rather than the medial prefrontal cortex, our results fit this model. This in turn explains why “cold” processes, for example, those related to the implementation of social norms such as generosity, are particularly affected.

Further studies are needed to investigate the exact correlations between impulsivity, cognitive control, generosity, and forgiveness. In this study, only data of highly impulsive participants were analyzed. In future studies, a healthy control group should also be included. This would make it possible to compare the effects of activating TBS on participants with different levels of greed, cognitive control and emotionality. Furthermore, investigating the role of other brain areas (e.g., the amygdala) in forgiveness processes in different groups of participants would be highly interesting. In addition, in further studies there should be a balance between female and male participants, as gender can have an influence on forgiveness processes (Women tend to be more forgiving, Buckholtz, 2015). The fact that the results contradict the initial hypothesis can be explained by the insufficiently large sample. In our power analysis, we assumed a large effect based on previous studies (e.g., Maier et al., 2018). In the present study, however, only a small to medium effect was shown. Future studies should therefore adjust the necessary number of participants according to a power analysis with a small to medium effect.

In summary, the results of this study were partly unexpected but provide interesting insights into the correlation between impulsivity and the effects of TBS. According to an initial interpretation of the results, the activating TBS of the right DLPFC was able to affect “cold” emotional processes but not “hot” emotions such as anger. Further research to clarify these results is needed.

## AUTHOR CONTRIBUTIONS

This study was designed by Moritz Maier, Martin Brüne and Ann‐Christine Ehlis. All measurements were performed by Moritz Maier and research assistants. Data correction and analyses were carried out by Moritz Maier and partly by David Rosenbaum under supervision of Ann‐Christine Ehlis. The manuscript was written by Moritz Maier and reviewed and complemented by Ann‐Christine Ehlis, Martin Brüne, David Rosenbaum and Andreas Fallgatter.
